# Supplementary material for: Paclitaxel and Caffeine–Taurine, New Colchicine Alternatives for Chromosomes Doubling in Maize Haploid Breeding
Source: Int J Mol Sci. 2023 Sep 28;24(19):14659. doi: 10.3390/ijms241914659 (PMC10572353; doi:10.3390/ijms241914659)
Supplement: Supplementary file 1 [file ijms-24-14659-s001.zip › ijms-2524823-supplementary.pdf]

International Journal of *Molecular Sciences*

Supplementary

Paclitaxel and Caffeine-Taurine, New Colchicine Alternatives for Chromosomes Doubling in Maize Haploid Breeding

Saeed Arshad[1], Mengli Wei[1], Qurban Ali[2], Ghulam Mustafa[2], Zhengqiang Ma[1] and Yuanxin Yan[1,3*]

[1]State Key Laboratory of Crop Genetics and Germplasm Enhancement, Nanjing Agricultural University, Nanjing 210095, China; maliksaeedawan@hotmail.com (S.A); ww1980666@126.com (ZM)
[2]Key Laboratory of Integrated Management of Crop Diseases and Pests, Ministry of Education, Department of Plant Pathology, College of Plant Protection, Nanjing Agricultural University, Nanjing 210095, China; rattarqurban@hotmail.com (Q.A); 2018201104@njau.edu.cn (G.M.).
[3]Jiangsu Collaborative Innovation Center for Modern Crop Production, Nanjing 210095, China
*Correspondence: yuanxin.yan@njau.edu.cn (Y.Y).

International Journal of *Molecular Sciences*

MDPI

Table S1. Comparative DH Seed Quantity Produced by Paclitaxel, Caffeine-Taurine and Colchicine by Seed Soaking Method

	Paclitaxel Seed Soaking Method						Colchicine Seed Soaking Method						Caffeine-Taurine Seed Soaking Method					
	**Treatment**	**RR (%)**	**OSR%**	**1-5 Seed/Ear**	**6-25 Seeds/Ear**	**>25 Seeds/Ear**	**Treatment**	**RR (%)**	**OSR%**	**1-5 Seed/Ear**	**6-25 Seeds/Ear**	**>25 Seeds/Ear**		**RR (%)**	**OSR%**	**1-5 Seed/Ear**	**6-25 Seeds/Ear**	**>25 Seeds/Ear**
**T1**	PTX 100 μM, 8h	0.0±0.0$^{g\,Z}$	0.0±0.0^{h}	0.0±0.0^{g}	0.0±0.0^{e}	0.0±0.0	Colchicine 0.5mM, 8h	2.3±0.7^{cde}	1.4±0.5^{cde}	2.3±0.3^{bc}	0.0±0.0^{c}	0.0±0.0	Caffeine 1g/L, 8h	3.9±1.0^{b}	2.5±0.5^{b}	3.9±1.0^{a}	0.0±0.0^{d}	0.0±0.0
**T2**	PTX 200 μM, 8h	0.7±0.3^{fg}	0.5±0.5^{gh}	0.7±0.2^{fg}	0.0±0.0^{e}	0.0±0.0	Colchicine 1.0mM, 8h	3.0±1.0^{cd}	1.9±0.6^{c}	3.0±1.0^{b}	0.0±0.0^{c}	0.0±0.0	Caffeine 2g/L, 8h	1.6±0.6^{cd}	1.0±1.0^{cd}	1.5±1.0^{b}	0.0±0.0^{d}	0.0±0.0
**T3**	PTX 400, 8h	0.0±0.0^{g}	0.0±0.0^{h}	0.0±0.0^{g}	0.0±0.0^{e}	0.0±0.0	Colchicine 1.5mM, 8h	1.4±0.4^{def}	1.0±0.5^{cde}	1.4±0.1^{cd}	0.0±0.0^{c}	0.0±0.0	Caffeine 4g/L, 8h	0.7±0.3^{de}	0.5±0.5^{de}	0.7±0.3^{bc}	0.0±0.0^{d}	0.0±0.0
**T4**	PTX 800 μM, 8h	1.5±0.6^{ef}	1.0±0.5^{efg}	1.5±0.3^{ef}	0.0±0.0^{e}	0.0±0.0	Colchicine 2.0mM, 8h	7.0±2.0^{a}	3.9±1.0^{a}	5.5±1.5^{a}	1.6±0.4^{a}	0.0±0.0	Caffeine 6g/L, 8h	2.4±0.6^{c}	1.4±0.4^{c}	1.6±0.4^{b}	0.8±0.2^{c}	0.0±0.0
**T5**	PTX 100 μM, 16h	5.3±1.2^{b}	3.3±0.7^{b}	3.8±0.4^{b}	1.5±0.4^{bc}	0.0±0.0	Colchicine 0.5mM, 16h	5.1±1.9^{b}	2.9±1.0^{b}	4.5±1.0^{a}	0.7±0.2^{b}	0.0±0.0	Caffeine 1g/L, 16h	6.0±2.0^{a}	3.9±0.6^{a}	3.8±0.9^{a}	1.5±0.5^{b}	0.7±0.3^{a}
**T6**	PTX 200 μM, 16h	2.4±0.4^{de}	1.5±0.5^{def}	2.4±0.7^{de}	0.0±0.0^{e}	0.0±0.0	Colchicine 1.0mM, 16h	3.5±1.0^{c}	1.6±0.4^{cd}	2.5±0.7^{bc}	0.8±0.3^{b}	0.0±0.0	Caffeine 2g/L, 16h	6.5±1.5^{a}	4.3±1.0^{a}	3.7±1.3^{a}	3.0±1.0^{a}	0.0±0.0
**T7**	PTX 400, 16h	0.8±0.8^{fg}	0.5±0.5^{gh}	0.8±0.4^{fg}	0.0±0.0^{e}	0.0±0.0	Colchicine 1.5mM, 16h	1.6±0.4^{def}	1.0±0.5^{cde}	1.6±0.6^{cd}	0.0±0.0^{c}	0.0±0.0	Caffeine 4g/L, 16h	0.0±0.0^{e}	0.0±0.0^{e}	0.0±0.0^{c}	0.0±0.0^{d}	0.0±0.0
**T8**	PTX 800 μM, 16h	3.5±1.0^{cd}	1.9±0.5^{cde}	2.6±0.9^{cd}	0.9±0.9^{d}	0.0±0.0	Colchicine 2.0mM, 16h	0.0±0.0^{f}	0.0±0.0^{f}	0.0±0.0^{e}	0.0±0.0^{c}	0.0±0.0	Caffeine 6g/L, 16h	0.0±0.0^{e}	0.0±0.0^{e}	0.0±0.0^{c}	0.0±0.0^{d}	0.0±0.0
**T9**	PTX 100 μM, 24h	7.8±1.3^{a}	4.8±0.8^{a}	5.5±1.5^{a}	2.3±0.5^{a}	0.0±0.0	Colchicine 0.5mM, 24h	1.6±0.5^{def}	0.9±0.3^{def}	1.6±0.4^{cd}	0.0±0.0^{c}	0.0±0.0	Caffeine 1g/L, 24h	0.0±0.0^{e}	0.0±0.0^{e}	0.0±0.0^{c}	0.0±0.0^{d}	0.0±0.0
**T10**	PTX 200 μM, 24h	3.3±0.7^{cd}	2.0±0.1^{cd}	3.3±0.3^{bcd}	0.0±0.0^{e}	0.0±0.0	Colchicine 1.0mM, 24h	1.5±0.5^{def}	1.0±0.1^{cde}	1.5±0.5^{cd}	0.0±0.0^{c}	0.0±0.0	Caffeine 2g/L, 24h	0.8±0.8^{de}	0.5±0.5d^{e}	1.0±0.2^{bc}	0.0±0.0^{d}	0.0±0.0
**T11**	PTX 400, 24h	5.6±1.4^{b}	3.0±1.0^{b}	3.7±0.3^{b}	1.9±0.1^{ab}	0.0±0.0	Colchicine 1.5mM, 24h	2.3±1.3^{cde}	1.4±0.5^{cde}	1.6±0.4^{cd}	0.8±0.5^{b}	0.0±0.0	Caffeine 4g/L, 24h	0.0±0.0^{e}	0.0±0.0^{e}	0.0±0.0^{c}	0.0±0.0^{d}	0.0±0.0
**T12**	PTX 800 μM, 24h	4.3±1.0^{bc}	2.4±1.0^{bc}	3.5±0.5^{bc}	1.0±0.5^{cd}	0.0±0.0	Colchicine 2.0mM, 24h	0.0±0.0^{f}	0.0±0.0^{f}	0.0±0.0^{e}	0.0±0.0^{c}	0.0±0.0	Caffeine 6g/L, 24h	0.0±0.0^{e}	0.0±0.0^{e}	0.0±0.0^{c}	0.0±0.0^{d}	0.0±0.0
**T13**	Control	0.7±0.3^{fg}	0.6±0.4^{fgh}	0.7±0.3^{fg}	0.0±0.0^{e}	0.0±0.0	Control	0.7±0.3^{ef}	0.6±0.4^{ef}	0.7±0.3^{de}	0.0±0.0^{c}	0.0±0.0	Control	0.7±0.3^{de}	0.6±0.4^{cde}	0.7±0.3^{bc}	0.0±0.0^{d}	0.0±0.0

Control = this treatment is identical to all other treatments but only ddH_2O was used; RR = reproduction rate; OSR = overall success rate; ^{Z} Mean ± SD; Different small letters on bars represent the significant differences within the treatments calculated using Tukey's HSD test at $p \leq 0.05$.

Table S2. Comparative DH Seed Quantity Produced by Paclitaxel and Colchicine by Seedling Immersion Method

	Paclitaxel Seedling Immersion Method						Colchicine Seedling Immersion Method					
	Treatment	RR (%)	OSR%	1-5 Seed/Ear	6-25 Seeds/Ear	>25 Seeds/Ear	Treatment	RR (%)	OSR%	1-5 Seed/Ear	6-25 Seeds/Ear	>25 Seeds/Ear
**T1**	PTX 100 μM, 8h	6.3±1.7$^{bcd\ Z}$	3.8±0.2^{bc}	6.3±1.3^{ab}	0.0±0.0^{c}	0.0±0.0^{b}	Colchicine 0.5mM, 8h	2.1±0.9^{ef}	1.5±0.5^{def}	2.1±0.4^{bcd}	0.0±0.0^{c}	0.0±0.0^{b}
**T2**	PTX 200 μM, 8h	5.1±1.5^{bcdef}	2.9±0.6^{bcde}	5.1±1.9^{bc}	0.0±0.0^{c}	0.0±0.0^{b}	Colchicine 1.0mM, 8h	3.0±0.5^{e}	2.0±0.5^{cd}	2.1±1.1^{bcd}	0.7±0.3^{bc}	0.0±0.0^{b}
**T3**	PTX 400, 8h	7.0±1.5^{b}	4.3±1.0^{ab}	6.2±1.3^{ab}	0.8±0.2^{b}	0.0±0.0^{b}	Colchicine 1.5mM, 8h	3.1±1.9^{de}	1.7±0.3^{de}	1.6±0.2^{d}	0.8±0.3^{bc}	0.8±0.4^{a}
**T4**	PTX 800 μM, 8h	4.3±1.0^{def}	2.0±1.0^{def}	3.5±0.5^{cd}	0.9±0.4^{b}	0.0±0.0^{b}	Colchicine 2.0mM, 8h	5.9±1.0^{ab}	2.5±0.5^{bcd}	4.0±1.0^{ab}	2.0±2.0^{a}	0.0±0.0^{b}
**T5**	PTX 100 μM, 16h	6.7±1.3^{bc}	3.8±0.8^{bc}	6.0±1.0^{ab}	0.8±0.2^{b}	0.0±0.0^{b}	Colchicine 0.5mM, 16h	5.6±1.4^{abc}	3.4±1.5^{ab}	4.0±2.0^{ab}	0.8±0.3^{bc}	0.8±0.2^{a}
**T6**	PTX 200 μM, 16h	6.5±1.5^{bcd}	3.8±0.4^{bc}	4.0±1.5^{cd}	1.6±0.4^{a}	0.8±0.4^{a}	Colchicine 1.0mM, 16h	5.3±1.0^{abcd}	3.0±1.0^{bc}	5.3±1.3^{a}	0.0±0.0^{c}	0.0±0.0^{b}
**T7**	PTX 400, 16h	9.3±1.8^{a}	5.7±1.0^{a}	7.8±1.3^{a}	1.6±0.4^{a}	0.0±0.0^{b}	Colchicine 1.5mM, 16h	5.6±1.0^{abc}	2.3±0.8^{bcd}	4.0±2.0^{ab}	1.7±0.3^{ab}	0.0±0.0^{b}
**T8**	PTX 800 μM, 16h	6.5±1.6^{bc}	3.3±0.8^{bcd}	4.0±1.0^{cd}	1.6±0.4^{a}	0.8±0.4^{a}	Colchicine 2.0mM, 16h	5.2±1.8^{abcd}	2.0±0.5^{cd}	5.2±1.8^{a}	0.0±0.0^{c}	0.0±0.0^{b}
**T9**	PTX 100 μM, 24h	4.6±1.4^{cdef}	3.0±1.0^{bcde}	3.8±0.8^{cd}	0.0±0.0^{c}	0.8±0.3^{a}	Colchicine 0.5mM, 24h	7.0±2.0^{a}	4.4±0.5^{a}	4.6±0.9^{a}	2.3±0.8^{a}	0.0±0.0^{b}
**T10**	PTX 200 μM, 24h	4.0±0.8^{ef}	2.4±0.6^{cde}	4.0±1.0^{cd}	0.0±0.0^{c}	0.0±0.0^{b}	Colchicine 1.0mM, 24h	3.7±1.0^{cde}	2.0±1.0^{cd}	3.7±0.9^{abc}	0.0±0.0^{c}	0.0±0.0^{b}
**T11**	PTX 400, 24h	5.2±1.3^{bcde}	3.0±2.0^{bcde}	3.4±0.6^{cd}	1.0±0.1^{b}	1.0±0.5^{a}	Colchicine 1.5mM, 24h	4.0±1.0^{bcde}	1.4±0.5^{def}	4.0±1.0^{abc}	0.0±0.0^{c}	0.0±0.0^{b}
**T12**	PTX 800 μM, 24h	3.0±1.0^{f}	1.6±0.4^{ef}	2.3±0.7^{de}	0.8±0.4^{b}	0.0±0.0^{b}	Colchicine 2.0mM, 24h	2.0±2.0^{ef}	0.5±0.5^{f}	2.0±1.0^{cd}	0.0±0.0^{c}	0.0±0.0^{b}
**T13**	Control	0.7±0.3^{g}	0.6±0.4^{f}	0.7±0.3^{e}	0.0±0.0^{c}	0.0±0.0^{b}	Control	0.7±0.3^{f}	0.6±0.4^{ef}	0.7±0.3^{d}	0.0±0.0^{c}	0.0±0.0^{b}

Control = this treatment is identical to all other treatments but only ddH_2O was used; RR = reproduction rate; OSR = overall success rate; ^{Z} Mean ± SD; Different small letters on bars represent the significant differences within the treatments calculated using Tukey's HSD test at $P \leq 0.05$.

International Journal of
Molecular Sciences

MDPI

Table S3. Number of Silks/Ear (V1).

REPLICATION	PLANT NO.	PACLITAXEL	COLCHICINE	CONTROL
R1	**P1**	235	190	255
R1	**P2**	240	199	272
R1	**P3**	236	210	260
R1	**P4**	255	208	272
R1	**P5**	240	225	269
R2	**P6**	230	211	278
R2	**P7**	233	200	252
R2	**P8**	223	194	260
R2	**P9**	226	202	278
R2	**P10**	247	217	271
R3	**P11**	243	209	280
R3	**P12**	235	222	263
R3	**P13**	230	220	275
R3	**P14**	230	192	252
R3	**P15**	250	209	262
R4	**P16**	241	219	275
R4	**P17**	220	223	274
R4	**P18**	238	197	278
R4	**P19**	220	220	261
R4	**P20**	229	192	260

Table S4. Number of Silks/Ear (V2).

REPLICATION	PLANT NO.	PACLITAXEL	COLCHICINE	CONTROL
R1	**P1**	251	208	263
R1	**P2**	265	220	280
R1	**P3**	270	175	298
R1	**P4**	247	210	267
R1	**P5**	263	225	277
R2	**P6**	266	195	320
R2	**P7**	240	229	318
R2	**P8**	259	210	317
R2	**P9**	247	235	295
R2	**P10**	240	211	305
R3	**P11**	265	222	320
R3	**P12**	267	228	315
R3	**P13**	255	223	289
R3	**P14**	259	227	309
R3	**P15**	249	236	305
R4	**P16**	263	192	289
R4	**P17**	262	199	311
R4	**P18**	270	195	290
R4	**P19**	238	230	319
R4	**P20**	227	232	292

Table S5. Plant Weight (V1).

REPLICATION	PLANT NO.	PACLITAXEL (gm)	COLCHICINE (gm)	CONTROL (gm)
R1	**P1**	107.254	80.641	130.257
R1	**P2**	105.540	82.514	129.954
R1	**P3**	100.920	72.980	138.500
R1	**P4**	95.520	75.541	140.451
R1	**P5**	97.254	84.654	137.890
R2	**P6**	99.652	80.325	143.540
R2	**P7**	100.612	82.540	140.980
R2	**P8**	105.420	63.920	147.540
R2	**P9**	109.525	66.528	138.000
R2	**P10**	101.786	69.653	133.654
R3	**P11**	108.845	81.958	140.985
R3	**P12**	104.654	85.560	143.571
R3	**P13**	101.520	84.120	140.100
R3	**P14**	108.000	83.369	131.540
R3	**P15**	110.110	78.800	141.240
R4	**P16**	105.520	75.970	134.620
R4	**P17**	107.542	76.652	137.210
R4	**P18**	97.980	77.980	139.328
R4	**P19**	98.200	82.641	141.500
R4	**P20**	97.328	81.890	142.900

Table S6. Plant Weight (V2).

REPLICATION	PLANT NO.	PACLITAXEL (gm)	COLCHICINE (gm)	CONTROL (gm)
R1	**P1**	123.100	105.580	140.120
R1	**P2**	119.256	99.870	147.322
R1	**P3**	119.900	107.210	150.125
R1	**P4**	118.230	103.560	155.142
R1	**P5**	120.541	97.520	160.245
R2	**P6**	121.580	105.900	155.456
R2	**P7**	116.400	102.240	151.258
R2	**P8**	117.528	99.230	150.140
R2	**P9**	116.800	101.250	147.890
R2	**P10**	119.485	100.510	150.548
R3	**P11**	120.250	97.650	152.254
R3	**P12**	125.854	102.451	156.230
R3	**P13**	123.950	103.215	158.650
R3	**P14**	117.300	106.542	160.210
R3	**P15**	119.350	102.420	143.654
R4	**P16**	117.200	105.120	147.700
R4	**P17**	122.850	107.621	147.982
R4	**P18**	130.740	101.250	152.200
R4	**P19**	124.250	100.321	145.650
R4	**P20**	121.300	105.510	144.255

Table S7. Genetic Materials and Experimental Locations.

Experiment #	Geno type Code	Germplasm Nature	Combination/Cross	Inducer Nature	Induction (season & year)	Experiment Locations	Year & Season of Study
Experiment 1 (PTX Field Experiment) Experiment 2 (CAF-T Field Experiment) Experiment 3 (Colchicine Field Experiment)	-	Tropical	((GO927 × 986) × Stock 6 Inducer)	Temperate	Spring-2020	Agriculture Research Station, Liuhe, JAAS	Spring-2022
Experiment 4* & 5*	V1	Temperate	((G3 × DH1667) × Stock 6 Inducer)	Temperate	Autumn-2019	Teaching and Research Centre, Baima, NJAU	Auumn-2022
	V2	Temperate	((PH6WC × O849) × Stock 6 Inducer)	Temperate			
Experiment 6* & 7* (Microscopic Validation)	-	Temperate	(((PH6WC × SD375) × SD375) × SD375) × Stock 6 Inducer))	Temperate	Spring-2020	NJAU	Autumn-2022

*Experiment 4 = Large scale studies; *Experiment 5 = Morphological and physiological studies; *Experiment 6 = PTX microscopic validation; *Experiment 7 = CAF-T microscopic validation; JAAS = Jiangsu Academy of Agricultural Sciences; NJAU = Nanjing Agricultural University
